# PATRIOT: A Pipeline for Tracing Identity-by-Descent for Chromosome Segments to Improve Genomic Prediction in Self-Pollinating Crop Species

**DOI:** 10.3389/fpls.2021.676269

**Published:** 2021-09-29

**Authors:** Johnathon M. Shook, Daniela Lourenco, Asheesh K. Singh

**Affiliations:** ^1^ Department of Agronomy, Iowa State University, Ames, IA, United States; ^2^ Department of Animal and Dairy Science, University of Georgia, Athens, GA, United States

**Keywords:** genomic selection, identity by descent, soybean, chromosomal tracing, genomic prediction

## Abstract

The lowering genotyping cost is ushering in a wider interest and adoption of genomic prediction and selection in plant breeding programs worldwide. However, improper conflation of historical and recent linkage disequilibrium between markers and genes restricts high accuracy of genomic prediction (GP). Multiple ancestors may share a common haplotype surrounding a gene, without sharing the same allele of that gene. This prevents parsing out genetic effects associated with the underlying allele of that gene among the set of ancestral haplotypes. We present “Parental Allele Tracing, Recombination Identification, and Optimal predicTion” (i.e., PATRIOT) approach that utilizes marker data to allow for a rapid identification of lines carrying specific alleles, increases the accuracy of genomic relatedness and diversity estimates, and improves genomic prediction. Leveraging identity-by-descent relationships, PATRIOT showed an improvement in GP accuracy by 16.6% relative to the traditional rrBLUP method. This approach will help to increase the rate of genetic gain and allow available information to be more effectively utilized within breeding programs.

## Introduction

Crop domestication has caused extreme genetic bottleneck, with a reduction in genetic diversity in domesticated crops compared to wild ancestors including in soybean (*Glycine max* L. Merr.; Hyten et al., 2006). Consequently, the number of ancestral individuals that are represented in modern cultivars is quite low (Gizlice et al., 1994). For example, 17 founding lines contributed 75% of the genes in modern US soybean cultivars, and 95% of genes could be traced to 35 ancestral lines, demonstrating an extremely narrow genetic variation challenging breeding progress. This is not confined to soybean alone, as other crops have similar challenges (Smith, 2007; Bennett et al., 2012).

The narrow genetic variability within modern breeding programs is a concern for breeders, as low diversity implies an incomplete sampling of favorable alleles as breeders attempt to improve crop performance and plasticity (Kisha et al., 1998). Furthermore, the likelihood of untapped resistance to biotic and abiotic stresses and the unavailability of favorable genes is high (Burdon, 2001). Low genetic diversity also negatively influences the response to selection (Tanksley and McCouch, 1997). In soybean, the continuous use of the same resistance source, i.e., PI 88788, has led to SCN populations developing increased reproduction on soybean varieties with this source, thereby necessitating additional sources of resistance in varieties (Tylka, 2007). Tracking identity-by-descent (IBD) presents unique advantages that can benefit ongoing plant breeding efforts in utilizing the narrow genetic germplasm pool within modern varieties effectively, as the limited number of founder sources increases the occurrence rate of each chromosomal segment from each founder. Each founder’s chromosomal segment is therefore expected to be replicated sufficiently within breeding materials to obtain accurate predictions of the segment effect.

Genomic selection (GS) is becoming mainstream in mid- to large breeding programs (Hickey et al., 2017), as it unlocks new opportunities to select in early generations and predict parental suitability (Battenfield et al., 2016; Yao et al., 2018). This leads to the ability to select improved lines accurately with less field testing and speed their reuse as parents in a breeding program. Such practice was only possible after the development of high-density marker panels that are currently available for many crops. Markers are widely used to infer relationships at the QTL level, which can be well estimated whether the LD between markers and QTL is reasonably high (Habier et al., 2007). Within breeding populations, markers can be expressed as either identical-by-state (IBS; individuals share nucleotide sequence; marker allele is the same independent of the origin) or IBD (individuals share nucleotide sequence; marker allele is the same by inheritance from a shared ancestor; Lynch and Walsh, 1998). IBD data provide greater information than IBS, as the nucleotide sequence between two adjacent IBD marker alleles from one parent in an individual is inherited from that same parent at a high probability, barring mutation or double recombination. When recombination is low within a region of multiple marker loci, it becomes possible to identify haplotypes, or runs of multiple markers which are consistently inherited together (Daly et al., 2001).

Current genomic selection models are predominantly based on IBS relationships between lines and utilize historic LD between markers and the trait of interest, as well as pedigree-based relatedness (Habier et al., 2007; Endelman, 2011). Modifications to the basic rrBLUP/GBLUP methods have had some success; for example, the SNP effects obtained in any SNP-based model can be converted into SNP variance and used as weights in genomic relationship-based models (Tiezzi and Maltecca, 2015). An extension to this model has also been proposed that accounts for heteroskedasticity (Shen et al., 2013). The basic approach has worked reasonably well in plants (Sorrells, 2015) and animals (VanRaden, 2008), which implies that IBS relationships are a reasonable approximation of the true IBD state. Where LD is high locally, IBS relationships are more similar to those calculated based on IBD. In other circumstances, the use of IBD can improve relationship estimation when compared to IBS (Li et al., 2014), can better account for population structure (Morrison, 2013), and can enhance genetic mapping (Dawn Teare and Barrett, 2005). Luan et al. (2012) compared IBS and IBD relationships for the estimation of genomic predictions in dairy cattle and found slightly greater additive genetic variance and accuracy for models based on IBD. Forneris et al. (2016) found IBD relationships to be more precise than IBS in simulated and real pig datasets; however, the authors reported that the computing time and memory needed to fit the hidden relatedness (i.e., IBD relationships through LD information) were high. This is because the method requires tracing IBD-inherited haplotypes within the pedigree (Thompson, 2013). The haplotype information from IBD due to inheritance from a recent common ancestor can therefore enable more accurate relationship estimates and improve the effectiveness of genomic selection with IBD-based genomic selection approaches. However, to take full advantage of the benefits of IBD data, it is necessary to track true IBD segments within the population, which requires knowledge about the pedigree and genotypes.

While previous efforts have relied on using haplotypes based on observed LD between markers, we explore an alternative approach of tracking the parental source of each allele. Two main distinctions between the approaches should be noted: (1) our approach does not assume any previous evidence of haplotypes or LD, instead utilizing markers which could only have been inherited from exactly one of the direct parents to define IBD segments, and (2) individuals which would otherwise have the same estimated effect from a shared haplotype can now be assigned different estimated effects due to tracking exactly which ancestral line a haplotype was inherited from.

We test an approach hereafter named “Parental Allele Tracing, Recombination Identification, and Optimal predicTion” (PATRIOT) that utilizes raw marker data for tracking IBD inheritance of chromosome segments, enabling the rapid identification of lines carrying specific alleles, increasing the accuracy of genomic relatedness and diversity estimates, and improving genomic prediction and selection performance. Using the SoyNAM population (Song et al., 2017), which includes 39 parents crossed to a common parent and 5,176 recombinant inbred lines, we explored the effectiveness of GS with additional information conferred with IBD (i.e., through PATRIOT). We traced chromosome segments from parent to progeny, followed by the calculation of the mean phenotype of lines inheriting each SNP from a given parental source. The difference between the mean phenotype of each SNP source and the population mean were used in place of the raw marker data to allow the incorporation of IBD data into a GS pipeline.

## Materials and Methods

### Pedigree Records

Pedigrees for public breeding lines tested in the Uniform Soybean Tests were recorded based on reporting in their last year of testing in the Northern tests^1^ or Southern tests.^2^ Additional breeding records were obtained from cultivar release papers, primarily from Crop Science,^3^ the Journal of Plant Registrations,^4^ and Canadian Journal of Plant Science.^5^ Pedigree information for other lines in the NPGS soybean germplasm collection were downloaded from https://npgsweb.ars-grin.gov/gringlobal/search. The pedigree information used in this study is provided in Supplementary File 1 and is also available from GitHub.^6^

### Marker Data

#### Soybean Nested Association Mapping Panel

**Figure 1:**
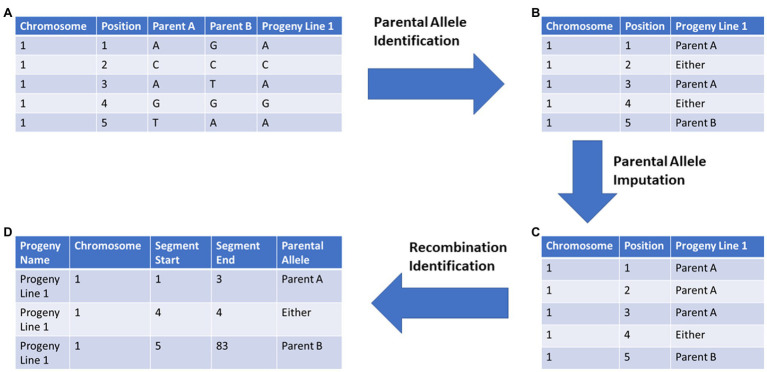
General workflow of Parental Allele Tracing, Recombination Identification, and Optimal predicTion (PATRIOT) input feature preparation for implementation in genomic selection: **(A)** Raw marker data are provided for both parent and progeny genotypes, **(B)** parental alleles encoded for those markers which can be conclusively traced to a specific parent, **(C)** alleles previously not assigned to a specific parent are imputed based on flanking markers, **(D)** those chromosome segments identical-by-descent from each parent are compiled. The “Position” column refers to the marker order and is provided only for demonstration purposes.

SNP marker data for 5,149 soybean nested association mapping (SoyNAM) RILs, as well as their parents, were downloaded from SoyBase,^7^ using the Wm82.a2 reference genome for downloading. For the SoyNAM panel, 4,289 SNP markers were used in the analysis. Markers were reordered prior to tracing and imputation based on the composite linkage map created in previous work (Song et al., 2017). The ancestral source of each chromosome segment was identified using the pipeline illustrated in Figure 1 and described below.

#### Released Cultivars and Isolines

We identified 868 accessions within the National Plant Germplasm System (NPGS) soybean collection wherein both parent and progeny were genotyped with the SoySNP50k SNP set, including near-isogenic lines derived from backcrossing schema. SNP marker data for all accessions in the GRIN database were downloaded from Soybase.org^8^ as a VCF file, with positions annotated based on the Wm82.a2 reference genome. Preprocessing to remove SNPs aligned to scaffolds or the mitochondria left 42,080 SNP markers aligned to the Wm82.a2 reference genome and used in further analysis. Missing SNP data were imputed using Beagle 4.0 with default settings (Browning and Browning, 2007). This panel will be referred to as the “868/50K panel” for brevity.

### Performance Data

Phenotypic records for the SoyNAM recombinant inbred line mapping population were downloaded from SoyBase (see Footnote 7), including yield, plant height, lodging, oil, and protein. Replicated entries’ phenotypic records from within a single environment were used to calculate BLUP for those lines, while unreplicated entries were incorporated using the raw phenotypic values. The “Corrected Strain” column was used to connect phenotypes with genotypic records. Phenotypic records were available from 2011 (IL and NE), 2012 (IA, IL, IN, KS, MI, MO, NE, OH^1^, and OH^2^), and 2013 (IA, IL, IN, KS, and MO). Additionally, SoyNAM RIL provided by Dr. George Graef was used to evaluate the performance of individual gene tracking for several qualitative traits (G. Graef, personal communication).

*Phytophthora* root rot resistance ratings were queried from the National Plant Germplasm System^9^ for each of the ancestors of the modern cultivar “Rend” (Nickell et al., 1999). “Rend” was selected for demonstration of the multi-generation chromosome segment tracing code due to both parents and all four grandparents being genotyped with the same platform, as well as the major resistance gene segregating within the pedigree.

### PATRIOT Workflow and Code Development

PATRIOT workflow utilizes LD and haplotype in a novel way to improve genomic prediction. Specifically, this system allows for the tracing of chromosomal segments from the immediate parents to the offspring, and to trace chromosomal segments through multiple generations. The allele tracing code outputs can be used as inputs into a modified genomic evaluation code, wherein the ancestral allele source records are converted to numeric based on differences from the population’s phenotypic mean. Custom R scripts were developed to identify SNPs which could only come from one of the listed parents (hereafter “anchor markers,” Figures 1A,B), followed by imputation of SNPs of fixed markers based on surrounding anchor markers (Figure 1C). Code for identifying anchor markers, imputation, multi-generation tracing, and recombination zone identification are available as **Code 1**, **Code 2**, **Code 3**, and **Code 4**, respectively (see footnote 6). Genomic prediction was evaluated using rrBLUP in R with raw marker data and allele tracing alternatives **Code 5** (see footnote 6).

The workflow can be translated into the following algorithm:

1. Prepare pedigree file for all individuals under consideration (backcross-derived lines should be coded as though they originated from a single cross).
2. Prepare a master marker file for progeny and parents which have been genotyped with the same marker panel.
3. Within each progeny, identify markers which could only have been inherited from one of the parents. Name those markers by their parental source and rename the remaining markers as “Parent A and Parent B.”
4. Impute ambiguous markers if they are flanked on either side by alleles inherited from the same parent. This often requires going more than one marker away to get to a marker which is known to be inherited from a specific parent.

To allow the nominal data created in steps 1–4 to be utilized for genomic prediction in linear regression-based approaches (e.g., ridge regression BLUP or rrBLUP), we created what we call an allele effect estimator. This requires the addition of three extra steps (5–7):

1. For each marker position, calculate the difference between the average phenotype of lines which inherited that marker from each parent and the location mean.

If there are eight different sources of alleles at a specific locus, there will be eight different estimates (one for each source). This process needs to be repeated separately for every location and trait. However, the same file of ancestral allele sources can be used regardless of environment or trait. The difference between the average phenotype of lines containing a specific ancestral allele and the location mean is the allele effect estimate (AEE or *α*):

$$\alpha_{j}=\frac{\sum y_{ij}}{n}-\mu$$ (1)

where *α_j_* is the allele effect estimate for ancestral allele source *j*, *y_ij_* is the phenotype for the *i*th line containing the ancestral allele source *j*, *n* is the total number of lines which inherited ancestral allele *j*, and *μ* is the population phenotypic mean.

In this way, separate allele effect estimates are created for each parental source of an allele. For loci whose ancestral source could not be determined (i.e., the nearest traced marker on either side come from different parents), the average of the two parental allelic differential estimators were used. Since each AEE is generated in a separate calculation, the AEE value is not regressed toward the mean to account for multiple regression. Instead, these values replace the marker representation as an input to GS models that evaluate the performance of this new approach (Table 1). They allow for the use of many distinct ancestral haplotypes in linear regression-based models based on the sign and relative scale of the estimated haplotype effect.

1. Create a new matrix (AEE matrix) by replacing the parental source of each locus with the estimated AEE for that parent at that locus. Markers for which parentage could not be differentiated are replaced with the average AEE of the two possible parents at that locus.
2. Within the context of genomic selection, replace the raw marker file (traditionally 0,1,2 or −1,0,1 format) with the AEE matrix (numeric matrix with positive and negative values, not restricted to integers).

**Table 1:** Simplified matrix showcasing parents, five potential progenies, and their AEEs.

	SNP 1	SNP 2	SNP 3	SNP 4	SNP 5	SNP 6	SNP 7	Prediction
Parent 1	45	−23	70	14	−56	73	15	+116.2
Parent 2	−40	17	−65	−50	−15	−51	70	−107.8
Parent 3	−53	20	−71	106	69	−36	−43	−4.5
Progeny 1	45	−23	70	−50	−15	−51	70	+34.7
Progeny 2	−40	17	−65	14	−56	73	15	−33.3
Progeny 3	−40	17	70	14	−56	73	15	+80.1
Progeny 4	45	17	70	14	−15	73	70	+249.6
Progeny 5	45	20	70	106	69	73	70	+431.1

AEEs were calculated using the full panel (more than one family) and with unequal population sizes, so allele effects are not necessarily equal and opposite. Progeny 2 and 3 differ based on the site of recombination around SNP 3. Progeny 4 represents the optimal combination of ancestral alleles available from within that population (Parent 1×Parent 2), but not necessarily within the full panel. Progeny 5 represents the global optimum progeny from within the panel. AEE scale is shown based on potential values for yield in terms of kg ha^−1^ in soybean.

### Chromosomal Tracing and Identity by Descent

As a proof of concept, tracing of chromosome segment inheritance within the pedigree of soybean cultivar “Rend” was performed. After ensuring consistency between expected results and the outputs, chromosome tracing was performed on the remainder of the 868/50K panel. Following completion of the single-generation tracing pipeline, the multi-generation tracing script was run on traced lines to allow visualization of multiple generations of inheritance and recombination.

In addition to the 868/50K panel, SoyNAM project parents and RILs were investigated with the chromosome tracing pipeline. The A/B genotype representation data available from SoyBase were utilized to impute chromosomal segments. Even with a sparse marker coverage, recombination events were still identifiable (Supplementary File 2). For SoyNAM families segregating for the known genes underlying the *T*, *I*, *R*, *W1*, and *Dt2* loci, those lines for which the immediate flanking markers were assigned to the same parental allele source were used to evaluate the accuracy of allele calling with PATRIOT IBD tracking.

### Genomic Prediction Models

To expand on the usefulness of the chromosome tracing pipeline outlined in Figure 1, we used the SoyNAM panel to evaluate accuracy of genomic prediction using ancestral alleles. Genomic prediction was evaluated for multiple traits (yield, moisture, oil, protein, fiber, lodging, days to maturity, and 100 seed weight) using the 39 SoyNAM RIL populations based on the phenotypic records available from the SoyNAM project and all 4,289 available markers. All comparisons were made using 80% of individuals phenotyped for the trait of interest in each environment for training and predicting on the remaining 20% of individuals.

Traditional rrBLUP performance was evaluated using mixed.solve, a function in R package “rrBLUP” (Endelman, 2011). The rrBLUP-PATRIOT analysis was performed using mixed.solve, but replacing the marker input data (0,1,2) with a matrix of AEEs calculated in PATRIOT. The mean observed phenotype of lines with top 10% of predicted performance using rrBLUP and PATRIOT were compared, as well as the difference in phenotype between selected lines and the base population. For yield, 5-fold cross-validation was used to reduce sampling bias in the estimation of GP accuracy for each method.

The performance of PATRIOT and rrBLUP was evaluated with *via* two approaches. For the first approach, we measure the correlation between predicted phenotypes and the observed phenotype in the testing set (lines not used to train the model). Improvement in genomic prediction accuracy was calculated by dividing the correlation between observed and predicted values using PATRIOT by the correlation between observed and predicted values using rrBLUP. In the second approach, we compared the mean phenotype of the testing lines with top 10% predicted phenotypes using PATRIOT and rrBLUP, and divide the mean of PATRIOT-selected lines by the mean of rrBLUP-selected lines to determine the improvement in genomic selection effectiveness. This second approach was then modified to compare the top 5% of lines for the 2012 OH^1^ yield test to gain further insight into where differences in model performance were most significant.

## Results

### Recombination Identification

For the 868/50Kpanel, 13.14% of all SNPs were unassigned to a specific parent. For the SoyNAM panel, 6.78% of all SNPs were unassigned to a specific parent. Using the SoyNAM panel marker data after PATRIOT IBD tracing and imputation, we examined the rates of recombination throughout the genome. Of the 5,149 RILs examined, we found total recombinations per line ranged from 10 to 557, with an average of 50.9 recombinations per line. The percentage of chromosomes that were inherited intact from one parent or another was 18.3% (18,808/102,960). A total of 5,011 RILs inherited at least one intact chromosome from a parent.

### Chromosomal Segment Tracing and Recombination Events

**Figure 2:**
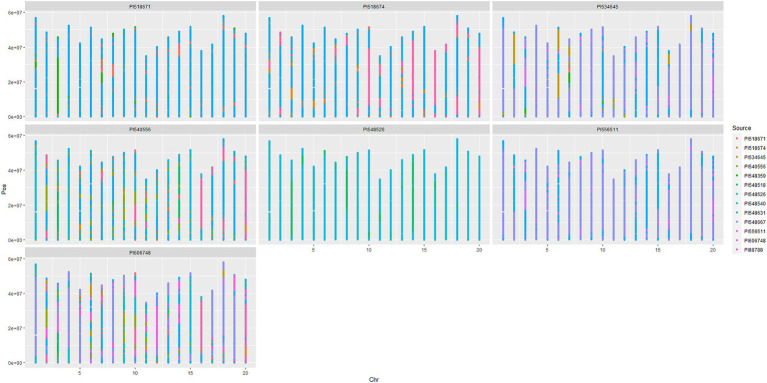
Scatterplot maps of chromosome segments inherited from ancestral sources, traced through progenitors of soybean cultivar Rend (i.e., PI606748). Chromosome number (based on Wms82.a2 reference genome) is plotted left to right on the x-axis, while position is plotted on the y-axis increasing bottom to top.

Chromosomal segments were traced in the 868/50K panel using the PATRIOT framework. To demonstrate the PATRIOT workflow, we traced the inheritance of the major *Phytophthora* root rot (PRR) resistance locus *Rps1* (Figure 2). Williams 82 (i.e., PI518671) inherited the *Rps1k* allele (that confers PRR resistance), as a long introgression (shown in green) on chromosome 3 from Kingwa (i.e., PI548359). This allele is then transmitted from Williams 82 to Resnik (i.e., PI534645) in a smaller chromosomal segment around *Rps1k*. However, the resistance allele was not passed on to Resnik’s progeny, Rend (i.e., PI606748). Resnik is therefore more suitable than Rend to breed for Phytophthora resistance. Chromosomal tracing over multiple generations allows presence/absence characterization for the *Rps1k* allele without the need for allele-specific markers and can reduce the need for phenotyping in disease nurseries, as allele state is known by virtue of IBD. Figure 2 gives a visual chromosomal segment tracing that is applicable to all varieties with available pedigree records that have been genotyped.

Recombination events can be visually identified when examining multiple generations within Figure 2 (or similar plots) in two ways using the chromosome 3 example: (i) between Williams 82 and Resnik, the length of the green segment surrounding *Rps1k* is greatly reduced in Resnik, indicating recombination during the cross of Asgrow 3127^4^×Williams 82, and (ii) a segment of the soft red “AmbiguousParentage” class appears in the progeny, which indicates that recombination occurs somewhere within this region, but could not be delimited between two adjacent markers due to multiple markers being alike by state in the parents. This occurs in Asgrow 3127 (i.e., PI556511) on chromosome 3, separating large segments inherited from Williams and Essex.

While the *Rps1k* example is provided, the PATRIOT framework is applicable to trace chromosomal regions and for IBD characterization of important genes through generations, as well as to visualize nearby recombination events. In addition, Table 2 provides a quick summary of the rate of concordance between allele calls and observed phenotypes.

**Table 2:** Rates of concordance between marker-based allele calls using flanking markers and the observed phenotype for five loci in the SoyNAM population.

Locus	Proportion of lines called correctly
*Dt2*	634/639 (99.2%)
*I*	532/544 (97.8%)
*R*	781/810 (96.4%)
*T*	1074/1136 (94.5%)
*W1*	2592/2651 (97.8%)

### Comparison of Genomic Prediction Accuracy Using SoyNAM

To examine the relative effectiveness of rrBLUP with PATRIOT (PATRIOT GS) compared to traditional rrBLUP (rrBLUP GS), yield predictions for 16 environments from each model were generated using the same randomized testing set for each model. Results from the two GS approaches are presented in Table 3. A 16.6% increase was attained in genomic prediction accuracy by using PATRIOT GS compared with traditional rrBLUP (0.557 vs. 0.478). Using a scenario of selecting 10% (and discarding 90%) from the SoyNAM RIL population and comparing to the overall SoyNAM RIL population mean, PATRIOT GS had an 8.6% greater selection differential among the selected RILs over basic rrBLUP GS (an increase of +538.7 in PATRIOT GS vs. +496.1kgha^−1^ in rrBLUP GS) Similar results were found for other traits, and can be found in Supplementary File 2.

**Table 3:** Comparison of the effectiveness of genomic selection methods rrBLUP GS and PATRIOT GS for yield.

Environment	Testing set mean	rrBLUP GS		PATRIOT GS		Marker-based heritability (h^2^)
	Testing set mean (kgha^−1^)	Average yield, top 10% (kgha^−1^)	^3^Correlation between observed phenotype and genomic prediction	Average yield, top 10% (kgha^−1^)	^3^Correlation between observed phenotype and genomic prediction	
2011 IL	2786.51 (12.26)	**3162.15 (59.99)**	0.51 (0.02)	3147.67 (59.58)	**0.53 (0.01)**	0.471
2011 NE	5048.98 (11.09)	**5538.78 (54.92)**	**0.62 (0.03)**	5420.83 (47.24)	0.57 (0.03)	0.614
2012 IA	2777.59 (10.38)	3263.39 (47.66)	0.43 (0.02)	**3313.28 (43.48)**	**0.51 (0.02)**	0.438
2012 IL	3390.61 (12.17)	3766.04 (75.62)	0.37 (0)	**3887.74 (45.01)**	**0.48 (0.02)**	0.415
2012 IN	4238.95 (12.81)	4761.52 (29.91)	0.5 (0.01)	**4773.01 (58.21)**	**0.56 (0.01)**	0.508
2012 KS	3875.14 (23.95)	4187.91 (39.67)	0.47 (0.03)	**4204.66 (33.05)**	**0.6 (0.02)**	0.548
2012 MI	2361.89 (32.5)	2785.12 (139.67)	0.57 (0.05)	**2849.09 (132.92)**	**0.73 (0.04)**	0.613
2012 MO	3458.43 (32.13)	4254.08 (65.28)	0.62 (0.04)	4244.01 (79.29)	**0.64 (0.03)**	0.603
2012 NE	4723.31 (18.08)	5251.94 (45.91)	0.44 (0.03)	**5288.3 (52.85)**	**0.51 (0.02)**	0.588
2012 OH^1^	3,402 (27.94)	3826.54 (133.36)	0.29 (0.04)	**3979.25 (167.09)**	**0.4 (0.05)**	0.285
2012 OH^2^	2811.38 (16.06)	3577.66 (99.49)	0.58 (0.04)	**3709.85 (103.35)**	**0.71 (0.04)**	0.664
2013 IA	2865.03 (15)	3216.05 (13.77)	0.39 (0.04)	**3266.87 (39.46)**	**0.47 (0.02)**	0.435
2013 IL	3113.94 (8.64)	3434.06 (33.06)	0.44 (0.03)	**3467.78 (28.75)**	**0.51 (0.02)**	0.459
2013 IN	5062.98 (17.78)	5492.83 (49.1)	0.38 (0.03)	**5521.03 (42.71)**	**0.45 (0.01)**	0.427
2013 KS	2759.03 (37.33)	3323.76 (67.07)	0.44 (0.02)	**3441.16 (63.83)**	**0.53 (0.01)**	0.548
2013 MO	4075.8 (11.47)	4848.04 (39.33)	0.61 (0.04)	**4855.79 (35.65)**	**0.71 (0.04)**	0.604

In each environment, the best model for each metric is highlighted in bold. Standard deviation given in parentheses. Each row contains results from a single site/year combination; “2011 IL” is therefore from an experiment in Illinois during 2011. IL, Illinois; NE, Nebraska; IA, Iowa; IN, Indiana; KS, Kansas; MI, Michigan; MO, Missouri; OH, Ohio.

1 and ^2^ represent two separate tests grown in Ohio in 2012.

3 Correlation between observed phenotype and Genomic Prediction is a measure of predictive ability or accuracy of prediction.

To help explain the cause of the difference in performance improvement between genomic prediction accuracy (+16.6%) and genomic selection effectiveness (+8.6%; both compared to rrBLUP), we further examined the yield predictions from the 2012 OH^1^ environment, which showed a large increase in GP accuracy (+39.5%) but only slight increase in genomic selection effectiveness (+3.8%). When examining the bottom 10% of predicted lines (rather than top 10% as before), the genomic selection effectiveness was 52.7% greater using PATRIOT than rrBLUP. This finding, coupled with smaller average absolute error terms using PATRIOT, suggests that the GP accuracy increase came from decreased error terms (PATRIOT prediction was closer to the observed phenotype than was rrBLUP prediction) throughout the full range of phenotypes, allowing for better rankings. Indeed, using a 5% selection level for high GEBVs using PATRIOT resulted in a 29.8% increase in average observed phenotype compared to rrBLUP in the 2012 OH^1^ set.

## Discussion

Some of the earlier efforts in soybean chromosomal tracing involved RFLP markers, as researchers traced chromosome segments in 67 genotypes through generations (Lorenzen, 1994). The transition to SNP markers as more mainstream marker technology enables better genome coverage to trace chromosomal segments from progenitors (Letcher and King, 2001), with increased resolution for recombination identification (Yu et al., 2011). However, the biallelic nature of SNP markers is a limitation for more refined haplotype generation. In the 868/50K panel, 13.14% of all markers could not be definitively traced back to their ancestral source. While some portion of this unassigned group can be attributed to heterozygous allele state in either one of the parents or the progeny, a substantial portion is due to recombination in the affected area in which both parents are IBS at several consecutive markers. A lower rate of singletons was found in the SoyNAM panel compared to the 868/50K panel.

The genome tracing of large segments through multiple generations enables breeders to follow genes of interest throughout the pedigrees of modern lines (Bruce et al., 2020). This allows for a rapid identification of lines containing the desired allele even if allele specific markers are not available. Visualization of relatedness of lines based on IBD metrics similar to what is shown in Figure 2 allows breeders to rapidly identify pairings of lines with high genetic diversity as parents to create breeding families (Liu and Anderson, 2003).

While IBD can be traced in many released public cultivars on the basis of markers from the SoySNP50K chip in soybean, applicability to breeding programs during the development of new pure lines requires a cost-effective genotyping system to allow genotyping of these lines at an earlier stage of development. This can be achieved by utilizing a smaller, less expensive genotyping array such as the SoyNAM6K BeadChip (Song et al., 2017) to genotype experimental lines.

The PATRIOT framework facilitates the identification of lines for breeding purposes that have favorable genes linked in coupling, as well as in situations where breaking the linkage drag is imperative. For example, SCN resistance from PI494182 was determined to carry a risk of linkage drag (St-Amour et al., 2020). Likewise, SCN resistance from the commonly used donor PI88788 was initially associated with considerable linkage drag (Cregan et al., 1999). With the use of PATRIOT, parents can be readily identified which contain the gene(s) of interest with the least amount of additional introgressed region(s), thereby reducing the likelihood of linkage drag, and concurrently deploy it in a GS pipeline. With an additional generation of traced progeny, those regions negatively associated with another trait can be identified to inform marker-based decisions.

Much like genome-wide association studies (GWAS), genomic prediction models rely on the association between markers and QTL. However, the association between marker and QTL decays in subsequent generations, leading to reduced accuracy without retraining of the model (Habier et al., 2007; Hayes et al., 2009; Jannink, 2010). With the chromosome tracing approach, the linkage between marker and QTL should withstand the decay better since parental allele representation is directly incorporated into the marker data. According to Li et al. (2005), when a SNP is in complete LD with a QTL or is at the QTL, this SNP provides sufficient information regarding the IBD state of a given locus. Based on that, the closer linkage between SNP and QTL among close relatives suggest that IBD relationships better reflect the similarity of individuals at the QTL level. This is because IBD is based on linkage generated by family structure, and relies on more recent generations, whereas IBS reflects relationships beyond pedigree recording (Luan et al., 2012).

The prediction accuracy is expected to decay much more slowly with chromosome tracing because the linkage between marker and QTL decays only when recombination occurs, rather than with changing founder allele frequency at a given locus. Furthermore, multi-generation tracing allows the preservation of information on lineage-specific marker association which can better model the differences in genes linked to a particular marker or set of markers. This concept can be elucidated with a hypothetical example with following conditions: (1) diploid organism, (2) single gene controlling the trait of interest, (3) trait of interest causes 1 unit increase in phenotype, (4) SNP marker is known and is 1cM away from the gene, and (5) wild population. In this scenario, the genetic information is given in Table 4.

**Table 4:** Hypothetical distribution of linkage between nearby marker and gene of interest.

Marker SNP	Gene	Percentage of population carrying the allele
A	Desired	0.30
A	Wild type	0.20
G	Desired	0.20
G	Wild type	0.30

With the incidence rate of the desirable allele, we can expect 0.5 unit phenotype level due to the causal gene. If the “A” allele of the SNP was selected, the total proportion selected will only be 50% but the phenotype level will only be 0.6units above the wild-type baseline. However, if the population was intermated after genotyping, and parental tracking for each progeny, marker–gene region can be tracked and therefore QTL effect can be accurately estimated by replication of the parental segment. These steps will ensure that with 1cM marker–gene linkage, the progeny after intermating can show 0.99 unit of phenotype level without model retraining.

This does not mean retraining or recalculation of SNP effects is not needed when IBD is used, but the decay in predictive ability is less. Other factors can also reduce the frequency of retraining. Hidalgo et al. (2021) showed that the decay in predictive ability was less when the number of genotyped individuals with phenotypes was greater than the number of independent chromosome segments (ICS). The ICS was defined as four times the effective population size (Ne) and the length of the genome in Morgans (Stam, 1980), which can be approached by the number of largest eigenvalues explaining 98% of the variance in the genomic relationship matrix (Pocrnic et al., 2016). Likewise, Luan et al. (2012) concluded that prediction accuracy based on IBD relationships were akin to those using IBS based on a higher-density SNP panel, and required only four generations of data without losses in accuracy.

According to Thompson (2013), if individuals share IBD segments from loci linked to the trait phenotypes, those individuals will have phenotypic similarities. Therefore, phenotypes provide information about the IBD state and pedigree relationships. The widespread use of PATRIOT GS would be encouraged by the establishment of a fully connected pedigree (fully known relationships between all germplasm utilized) and development of base population resources with equal and wide representation of each parental source within the breeding pool. For example, while the SoyNAM panel can be readily used as a training set for materials derived from any combination of the 40 parents, its efficacy is limited to that context, with the exception of a small number of the parents’ ancestors within the pool. Instead, in some situations, breeding applications would benefit from the development of fully interrelated populations derived from the original founder lines, such as through MAGIC design (Li et al., 2013; Dell’Acqua et al., 2015) or a NAM population created with founder parents (Yu et al., 2008) that can happen in different crossing cycles. Moreover, most breeding programs have an inherent nested design especially when a few superior parents are used extensively in the development of breeding populations, therefore this effort is not incremental.

The multi-generation chromosome segment tracing aspect of PATRIOT can also be used as a tool to connect QTL mapping studies among related populations. In addition to tracing chromosomal regions within a pedigree, this framework can be used to connect linkage mapping studies using related lines as parents by tracing QTL regions identified in related parents in separate studies to their ancestral sources. This allows for a meta-analysis to utilize the increased power which comes from having multiple mapping populations with common ancestry to map marker–trait associations.

However, there are challenges to the PATRIOT framework. In crosses where parents share large runs of IBS or IBD based on marker data, it is difficult to determine which parent is contributing each allele to the progeny. However, if these runs are IBD, the effect on allele estimation is equivalent, regardless of which parent is assigned to the allele. Additionally, a surprising number of singleton marker calls suggests that either double recombination is occurring at a much higher rate than previously believed, or that the reference genome assembly order does not agree with the true marker order. Increased marker density can overcome some of these challenges. Likewise, uncertain regions can be assigned new allele effect classes. For example, Williams 82 (PI518671) has 3,399 out of 42,080 markers which could not be assigned with certainty to a specific parent (Williams or Kingwa). To circumvent this challenge, each of these markers was assigned a new parent class of “PI518671” when tracking segments passed on to progeny but continue to use AEEs based on the average AEE of parents Williams and Kingwa when predicting its own performance.

PATRIOT genomic prediction accuracy for yield using all populations was greater than the calculated marker-based heritability of the trait in 13 of 16 environments (Table 3), suggesting that genomic prediction using ancestral allele tracing can perform better than traditional genomic prediction. Generating separate prediction models in this way for each environment may be explored as an avenue to reduce the number of environments needed for phenotypic evaluation, as the prediction accuracy very nearly reaches the heritability of the trait itself. Alternatively, a model trained on the whole target population of environments rather than a single environment can be developed to predict varieties that are expected to perform best across a wider range of environments.

The fact that this high level of prediction accuracy was possible with a 6K SNP chip in the SoyNAM populations suggest significant potential cost savings, as the cost of genotyping at this density is less expensive than growing and phenotyping in replicated field plots (Xu et al., 2020). More generally speaking, if small arrays are to continue to be used in community research projects, the array needs to be carefully designed to provide adequate coverage throughout the genome. Consideration of both linkage distance and optimal SNP selection in genic regions should be made a priority. Alternatively, other genotyping platforms such as genotyping-by-sequencing (GBS) can be used to implement this approach, which is able to decrease the negative impact of missing data that are common from GBS (Gardner et al., 2014).

While our genomic prediction models utilized only the immediate parents for calculating allele effect estimates, it is possible to expand the method by combining with the multi-generation IBD tracing script. This approach has an added benefit of bridging the gap between populations that do not share a direct parent but share ancestors in previous generations. By doing so, an increased number of lines can be used for allele effect estimation, further improving the accuracy of these values.

IBD-based genomic selection has the clear potential to improve selection accuracy over existing genomic selection approaches. However, there is a trade-off due to the significant increase in computational time (Forneris et al., 2016). While the chromosome segment tracing portion of the workflow need only be run once for any genotype, the AEE matrix must be calculated separately for each trait and environment. Fortunately, this calculation can be parallelized, and only needs to be performed for the training population. Typical computation time on an AMD Ryzen Threadripper 1950X for AEE matrix calculation was on the order of 1min without parallelization of the code, while the genomic prediction itself took on the order of 3min for a dataset with 2,500 individuals and 4,289 markers. Computation time for the tracing and imputation of alleles within the SoyNAM study totaled 7h 41min. However, minor modifications to run each chromosome in parallel on different computational threads has the potential to reduce the wall time to around 35min. Further studies are needed to determine the repeatability of the PATRIOT pipeline for IBD allele coding and genomic selection in the above-described scenarios.

## Conclusion

The PATRIOT pipeline provides a framework for identifying, tracking, and applying IBD information to increase effectiveness of genomic selection under SNP-based models. Tracking IBD with PATRIOT enables pedigree-based gene tracking through generations, which can be useful for parental selection, as well as for predicting phenotypes for monogenic and oligogenic traits. Relatedness metrics within breeding populations can also be improved due to the specification of IBD allele sharing rather than IBS. The IBD information also works to improve genomic prediction and selection results. This improvement was shown in first-cycle genomic prediction but should provide additional benefits in later cycles due to the donor-specific allele effect estimation, which does not suffer from the problem of population shift between training and testing sets. The large and consistent benefit shown suggests that chromosome tracing is a quick and efficient way to increase the accuracy of genomic selection models, with no additional cost beyond modestly increased computational time.

## Data Availability Statement

Publicly available datasets were analyzed in this study. These datasets can be found at: https://github.com/SoylabSingh/PATRIOT, https://soybase.org/SoyNAM/index.php, and https://soybase.org/snps/.

## Author Contributions

JS conceptualized the project with AS and conducted the statistical analysis with suggestions from AS and DL. JS and AS prepared the first draft. All authors contributed to the article and approved the submitted version.

## Funding

Authors sincerely appreciate the funding support from Iowa Soybean Association, R. F. Baker Center for Plant Breeding, Bayer Chair in Soybean Breeding, and USDA CRIS project (IOW04714). Part of JS graduate assistance was provided by the NSF NRT (graduate fellowship).

## Conflict of Interest

The authors declare that the research was conducted in the absence of any commercial or financial relationships that could be construed as a potential conflict of interest.

## Publisher’s Note

All claims expressed in this article are solely those of the authors and do not necessarily represent those of their affiliated organizations, or those of the publisher, the editors and the reviewers. Any product that may be evaluated in this article, or claim that may be made by its manufacturer, is not guaranteed or endorsed by the publisher.

## Footnotes

^1^ https://www.ars.usda.gov/midwest-area/west-lafayette-in/crop-production-and-pest-control-research/docs/uniform-soybean-tests-northern-region/

^2^ https://www.ars.usda.gov/southeast-area/stoneville-ms/crop-genetics-research/docs/uniform-soybean-tests/

^3^ https://acsess.onlinelibrary.wiley.com/journal/14350653

^4^ https://acsess.onlinelibrary.wiley.com/journal/19403496

^5^ https://www.nrcresearchpress.com/journal/cjps

^6^ https://github.com/SoylabSingh/PATRIOT

^7^ https://soybase.org/SoyNAM/index.php

^8^ https://soybase.org/dlpages/#snp50k

^9^ https://npgsweb.ars-grin.gov/gringlobal/search.aspx

## Abbreviations

GP: Genomic prediction
GS: Genomic selection
GEBV: Genomic Estimated Breeding Value
gBLUP: Genomic best linear unbiased predictors
rrBLUP: Ridge regression best linear unbiased predictors
PATRIOT: “Parental Allele Tracing, Recombination Identification, and Optimal predicTion”
IBD: Identity-by-descent
IBS: Identity-by-state
LD: Linkage disequilibrium
QTL: Quantitative trait locus
MAS: Marker-assisted selection
USDA: United States Department of Agriculture
NAM: Nested Association Mapping (population)
SNP: Single-nucleotide polymorphism

## References

[ref1] Battenfield S. D. Guzmán C. Gaynor R. C. Singh R. P. Peña R. J. Dreisigacker S.. (2016). Genomic selection for processing and end-use quality traits in the CIMMYT spring bread wheat breeding program. Plant Genome 9, 1–12. doi: 10.3835/plantgenome2016.01.0005, PMID: 27898810

[ref2] Bennett A. J. Bending G. D. Chandler D. Hilton S. Mills P. (2012). Meeting the demand for crop production: the challenge of yield decline in crops grown in short rotations. Biol. Rev. 87, 52–71. doi: 10.1111/j.1469-185X.2011.00184.x, PMID: 21631700

[ref3] Browning S. R. Browning B. L. (2007). Rapid and accurate haplotype phasing and missing-data inference for whole-genome association studies by use of localized haplotype clustering. Am. J. Hum. Genet. 81, 1084–1097. doi: 10.1086/521987, PMID: 17924348 PMC2265661

[ref4] Bruce R. W. Torkamaneh D. Grainger C. M. Belzile F. Eskandari M. Rajcan I. (2020). Haplotype diversity underlying quantitative traits in Canadian soybean breeding germplasm. Theor. Appl. Genet. 133, 1967–1976. doi: 10.1007/s00122-020-03569-1, PMID: 32193569

[ref5] Burdon R. D. (2001). Genetic diversity and disease resistance: some considerations for research, breeding, and deployment. Can. J. For. Res. 31, 596–606. doi: 10.1139/x00-136

[ref6] Cregan P. B. Mudge J. Fickus E. W. Danesh D. Denny R. Young N. D. (1999). Two simple sequence repeat markers to select for soybean cyst nematode resistance coditioned by the rhg1 locus. Theor. Appl. Genet. 99, 811–818. doi: 10.1007/s001220051300

[ref7] Daly M. J. Rioux J. D. Schaffner S. F. Hudson T. J. Lander E. S. (2001). High-resolution haplotype structure in the human genome. Nat. Genet. 29, 229–232. doi: 10.1038/ng1001-229, PMID: 11586305

[ref8] Dawn Teare M. Barrett J. H. (2005). Genetic linkage studies. Lancet 366, 1036–1044. doi: 10.1016/S0140-6736(05)67382-5 16168786

[ref9] Dell’Acqua M. Gatti D. M. Pea G. Cattonaro F. Coppens F. Magris G.. (2015). Genetic properties of the MAGIC maize population: a new platform for high definition QTL mapping in Zea mays. Genome Biol. 16, 167–167. doi: 10.1186/s13059-015-0716-z, PMID: 26357913 PMC4566846

[ref10] Endelman J. B. (2011). Ridge regression and other kernels for genomic selection with R package rrBLUP. Plant Genome 4, 250–255. doi: 10.3835/plantgenome2011.08.0024

[ref11] Forneris N. S. Steibel J. P. Legarra A. Vitezica Z. G. Bates R. O. Ernst C. W.. (2016). A comparison of methods to estimate genomic relationships using pedigree and markers in livestock populations. J. Anim. Breed. Genet. 133, 452–462. doi: 10.1111/jbg.12217, PMID: 27135179

[ref12] Gardner K. M. Brown P. Cooke T. F. Cann S. Costa F. Bustamante C.. (2014). Fast and cost-effective genetic mapping in apple using next-generation sequencing. G3 4:1681. doi: 10.1534/g3.114.011023, PMID: 25031181 PMC4169160

[ref13] Gizlice Z. Carter T. E. Jr. Burton J. W. (1994). Genetic base for North American Public soybean cultivars released between 1947 and 1988. Crop Sci. 34, 1143–1151. doi: 10.2135/cropsci1994.0011183X003400050001x

[ref14] Habier D. Fernando R. L. Dekkers J. C. M. (2007). The impact of genetic relationship information on genome-assisted breeding values. Genetics 177, 2389–2397. doi: 10.1534/genetics.107.081190, PMID: 18073436 PMC2219482

[ref15] Hayes B. J. Visscher P. M. Goddard M. E. (2009). Increased accuracy of artificial selection by using the realized relationship matrix. Genet. Res. 91, 47–60. doi: 10.1017/S0016672308009981, PMID: 19220931

[ref16] Hickey J. M. Chiurugwi T. Mackay I. Powell W. Eggen A. Kilian A.. (2017). Genomic prediction unifies animal and plant breeding programs to form platforms for biological discovery. Nat. Genet. 49, 1297–1303. doi: 10.1038/ng.3920, PMID: 28854179

[ref17] Hidalgo J. Lourenco D. Tsuruta S. Masuda Y. Miller S. Bermann M.. (2021). Changes in genomic predictions when new information is added. J. Anim. Sci. 99:skab004. doi: 10.1093/jas/skab004, PMID: 33544869 PMC7867035

[ref18] Hyten D. L. Song Q. Zhu Y. Choi I.-Y. Nelson R. L. Costa J. M.. (2006). Impacts of genetic bottlenecks on soybean genome diversity. Proc. Natl. Acad. Sci. 103, 16666–16671. doi: 10.1073/pnas.0604379103, PMID: 17068128 PMC1624862

[ref19] Jannink J.-L. (2010). Dynamics of long-term genomic selection. Genet. Sel. Evol. 42:35. doi: 10.1186/1297-9686-42-35, PMID: 20712894 PMC2936280

[ref20] Kisha T. J. Diers B. W. Hoyt J. M. Sneller C. H. (1998). Genetic diversity among soybean plant introductions and North American Germplasm. Crop Sci. 38, 1669–1680. doi: 10.2135/cropsci1998.0011183X003800060042x

[ref21] Letcher B. H. King T. L. (2001). Parentage and grandparentage assignment with known and unknown matings: application to Connecticut River Atlantic salmon restoration. Can. J. Fish. Aquat. Sci. 58, 1812–1821. doi: 10.1139/f01-125

[ref22] Li M. Boehnke M. Abecasis G. R. (2005). Joint modeling of linkage and association: identifying SNPs responsible for a linkage signal. Am. J. Hum. Genet. 76, 934–949. doi: 10.1086/430277, PMID: 15877278 PMC1196453

[ref23] Li H. Glusman G. Hu H. Shankaracharya Caballero J. Hubley R.. (2014). Relationship estimation from whole-genome sequence data. PLoS Genet. 10:e1004144. doi: 10.1371/journal.pgen.1004144, PMID: 24497848 PMC3907355

[ref24] Li X.-F. Liu Z.-X. Lu D.-B. Liu Y.-Z. Mao X.-X. Li Z.-X.. (2013). Development and evaluation of multi-genotype varieties of rice derived from MAGIC lines. Euphytica 192, 77–86. doi: 10.1007/s10681-013-0879-1

[ref25] Liu S. Anderson J. A. (2003). Marker assisted evaluation of fusarium head blight resistant wheat Germplasm. Crop Sci. 43, 760–766. doi: 10.2135/cropsci2003.7600

[ref26] Lorenzen L. L. (1994). Soybean Cultivar Development: A Genome Perspective. Doctor of Philosophy, Iowa State University.

[ref27] Luan T. Woolliams J. A. Ødegård J. Dolezal M. Roman-Ponce S. I. Bagnato A.. (2012). The importance of identity-by-state information for the accuracy of genomic selection. Genet. Sel. Evol. 44:28. doi: 10.1186/1297-9686-44-28, PMID: 22937985 PMC3517337

[ref28] Lynch M. Walsh B. (1998). Genetics and Analysis of Quantitative Traits. Sunderland, MA: Sinauer.

[ref29] Morrison J. (2013). Characterization and correction of error in genome-wide IBD estimation for samples with population structure. Genet. Epidemiol. 37, 635–641. doi: 10.1002/gepi.21737, PMID: 23740691 PMC4001853

[ref30] Nickell C. D. Noel G. R. Cary T. R. Thomas D. J. Hoffman D. D. (1999). Registration of ‘Rend’ soybean. Crop Sci. 39, 1533–1534. doi: 10.2135/cropsci1999.0012rcv

[ref31] Pocrnic I. Lourenco D. A. L. Masuda Y. Legarra A. Misztal I. (2016). The dimensionality of genomic information and its effect on genomic prediction. Genetics 203, 573–581. doi: 10.1534/genetics.116.187013, PMID: 26944916 PMC4858800

[ref32] Shen X. Alam M. Fikse F. Rönnegård L. (2013). A novel generalized ridge regression method for quantitative genetics. Genetics 193, 1255–1268. doi: 10.1534/genetics.112.146720, PMID: 23335338 PMC3606101

[ref33] Smith S. (2007). Pedigree pedigree background changes in U.S. hybrid maize between 1980 and 2004. Crop Sci. 47, 1914–1926. doi: 10.2135/cropsci2006.12.0763

[ref34] Song Q. Yan L. Quigley C. Jordan B. D. Fickus E. Schroeder S.. (2017). Genetic characterization of the soybean nested association mapping population. Plant Genome 10, 1–14. doi: 10.3835/plantgenome2016.10.0109, PMID: 28724064

[ref35] Sorrells M. E. (2015). “Genomic selection in plants: empirical results and implications for wheat breeding,” in Advances in Wheat Genetics: From Genome to Field. eds. Ogihara Y. Takumi S. Handa H. (Tokyo: Springer).

[ref36] Stam P. (1980). The distribution of the fraction of the genome identical by descent in finite random mating populations. Genet. Res. 35, 131–155. doi: 10.1017/S0016672300014002

[ref37] St-Amour V. T. B. Mimee B. Torkamaneh D. Jean M. Belzile F. O’Donoughue L. S. (2020). Characterizing resistance to soybean cyst nematode in PI 494182, an early maturing soybean accession. Crop Sci. 60, 2053–2069. doi: 10.1002/csc2.20162

[ref38] Tanksley S. D. McCouch S. R. (1997). Seed banks and molecular maps: unlocking genetic potential from the wild. Science 277, 1063–1066. doi: 10.1126/science.277.5329.1063, PMID: 9262467

[ref39] Thompson E. A. (2013). Identity by descent: variation in meiosis, across genomes, and in populations. Genetics 194, 301–326. doi: 10.1534/genetics.112.148825, PMID: 23733848 PMC3664843

[ref40] Tiezzi F. Maltecca C. (2015). Accounting for trait architecture in genomic predictions of us Holstein cattle using a weighted realized relationship matrix. Genet. Sel. Evol. 47:24. doi: 10.1186/s12711-015-0100-1, PMID: 25886167 PMC4381547

[ref41] Tylka G. (2007). “Current Status of the Soybean Cyst Nematode as a Threat to Soybean Production in the Midwest.” in *Integrated Crop Management Conference*; November 28–29, 2007.

[ref42] VanRaden P. M. (2008). Efficient methods to compute genomic predictions. J. Dairy Sci. 91, 4414–4423. doi: 10.3168/jds.2007-0980, PMID: 18946147

[ref43] Xu Y. Liu X. Fu J. Wang H. Wang J. Huang C.. (2020). Enhancing genetic gain through genomic selection: from livestock to plants. Plant Commun. 1:100005. doi: 10.1016/j.xplc.2019.100005, PMID: 33404534 PMC7747995

[ref44] Yao J. Zhao D. Chen X. Zhang Y. Wang J. (2018). Use of genomic selection and breeding simulation in cross prediction for improvement of yield and quality in wheat (*Triticum aestivum* L.). Crop J. 6, 353–365. doi: 10.1016/j.cj.2018.05.003

[ref45] Yu J. Holland J. B. McMullen M. D. Buckler E. S. (2008). Genetic design and statistical power of nested association mapping in maize. Genetics 178, 539–551. doi: 10.1534/genetics.107.074245, PMID: 18202393 PMC2206100

[ref46] Yu H. Xie W. Wang J. Xing Y. Xu C. Li X.. (2011). Gains in QTL detection using an ultra-high density SNP map based on population sequencing relative to traditional RFLP/SSR markers. PLoS One 6:e17595. doi: 10.1371/journal.pone.0029633, PMID: 21390234 PMC3048400

